# Changes in active DNA demethylation pathway components during neoadjuvant chemotherapy and their prognostic significance in breast cancer

**DOI:** 10.1186/s13058-026-02314-8

**Published:** 2026-06-18

**Authors:** Jolanta Guz, Olga Urbanowska-Domanska, Ewelina Zarakowska, Daniel Gackowski, Rafal Rozalski, Kinga Linowiecka, Aleksandra Skalska-Bugala, Piotr Rhone, Ryszard Olinski, Marek Foksinski

**Affiliations:** 1 Department of Clinical Biochemistry, Faculty of Pharmacy, Collegium Medicum in Bydgoszcz, Nicolaus Copernicus University in Toruń, Karlowicza 24, 85‑092 Bydgoszcz, Poland; 2 Department of Oncology, Professor Franciszek Lukaszczyk Oncology Centre, Romanowskiej 2, 85-796 Bydgoszcz, Poland; 3 Department of Human Biology, Institute of Biology, Faculty of Biological and Veterinary Sciences, Nicolaus Copernicus University in Toruń, Lwowska 1, 87-100 Toruń, Poland; 4 Department of Immunology, Faculty of Pharmacy, Collegium Medicum in Bydgoszcz, Nicolaus Copernicus University in Toruń, M. Skłodowskiej Curie 9, 85-094 Bydgoszcz, Poland; 5 Clinical Ward of Breast Cancer and Reconstructive Surgery, Professor Franciszek Lukaszczyk Oncology Centre, Romanowskiej 2, 85-796 Bydgoszcz, Poland

**Keywords:** Active DNA demethylation, Breast cancer, Neoadjuvant chemotherapy, Ki-67

## Abstract

**Background:**

Neoadjuvant chemotherapy (NACT) for early breast cancer is a systemic treatment administered before surgery to achieve regression of the primary lesion, perform breast-conserving surgery, if possible, and ensure long-term recurrence-free survival. The main aim of this study was to investigate the impact of NACT on the active DNA demethylation process and the relationship between the compounds involved in this path and disease-free survival (DFS) and overall survival (OS) during a six-year follow-up.

**Methods:**

This study included 71 patients with breast cancer who were eligible for NACT consisting of 4 cycles of doxorubicin and cyclophosphamide regimens at doses of 60 mg/m^2^ and 600 mg/m^2^, respectively, on the first day every 21 days, as well as paclitaxel administered at a dose of 80 mg/m^2^ weekly for 12 weeks. Peripheral blood and urine samples were collected from breast cancer patients at four time points. Additionally, tissue samples from a breast cancer tumor and regional metastatic lymph nodes were collected during surgery. The levels of 5-methylcytosine (5-mCyt) and its derivatives in DNA and urine were determined via two-dimensional ultra-performance liquid chromatography with tandem mass spectrometry (2D-UPLC-MS/MS). The qRT‒PCR technique was used to determine the transcript levels of the genes involved in active DNA demethylation.

**Results:**

During chemotherapy, increases in the levels of 5-methyl-2ʹ-deoxycytidine (5-mdC), 5-formyl-2ʹ-deoxycytidine (5-fdC), and 5-carboxyl-2ʹ-deoxycytidine (5-cadC) and decreases in the expression of genes encoding TET (ten-eleven translocation) proteins, TDG (thymine DNA glycosylase), and AID (activation-induced cytidine deaminase) were observed in peripheral blood leukocytes. The Kaplan‒Meier curves revealed that patients with a lower level of 5-mdC in leukocyte DNA and urine before NACT, higher levels of 5-cadC, and lower *TDG* expression in tumor cells after treatment had a more favorable prognosis. Furthermore, significant correlations were found between the levels of 5-fdC in the DNA of leukocytes, 5-cadC in DNA tumor cells, the expression of *TET2*, and decreased Ki-67 after NACT.

**Conclusions:**

Our results indicate that NACT can alter active DNA demethylation markers. Breast cancer patients with prolonged DFS had lower leukocyte 5-mdC and higher urinary 5-mdC levels before chemotherapy. Thus, assessing 5-mdC in these minimally invasive biospecimens may provide prognostic information for breast cancer patients undergoing NACT.

## Background

Breast cancer is the most frequently diagnosed malignant tumor among women worldwide. The incidence of this type of cancer is constantly increasing worldwide [1]. Current methods for treating breast cancer include surgery and systemic treatment, such as chemotherapy, immunotherapy, hormone therapy, and radiotherapy. The order and choice of the treatment methods mentioned above depend strictly on the tumor’s prognostic and predictive factors, the disease’s stage, the patient’s general condition, and concomitant diseases [2]. The qualification of patients for appropriate systemic treatment is based on fundamental histopathological and immunohistochemical tests [3]. However, in the era of personalized medicine, there remains a critical need to identify new predictive factors in breast cancer patients to develop more tailored treatment approaches, potentially improving patient prognoses [4].

Neoadjuvant chemotherapy (NACT) is a systemic treatment administered to breast cancer patients before surgical treatment to improve surgical options and obtain long-term disease-free survival (DFS) [5]. The indications for NACT are locally advanced breast cancer and inflammatory breast cancer. NACT is also the standard treatment for biologically aggressive subtypes of breast cancer at lower stages of local advancement, particularly in human epidermal growth factor receptor 2 (HER2)-positive and triple-negative breast cancer, owing to the extremely high risk of disease recurrence, characterized by distant metastasis [6, 7]. Despite well-defined prognostic factors such as age, local advancement, degree of malignancy, and known predictive factors, including hormonal receptor expression and HER2 receptor overexpression [8], disease recurrence is observed in approximately 25–30% of patients [9]. Therefore, there is a need to identify new, more sensitive, and personalized predictive factors.

Aberrant DNA methylation is a common hallmark of carcinogenesis and has been documented in breast cancer. These abnormal methylation patterns can be detected at early stages of disease development and have significant implications for tumor progression and metastasis [10–13], as well as being closely associated with patient prognosis [11, 14]. DNA methylation is an epigenetic process that modulates gene expression and chromatin structure [15]. In turn, the most dynamic process that can affect DNA methylation patterns is active demethylation involving TET (Ten-eleven translocation) proteins and AID (Activation-Induced Cytidine Deaminase) [16]. The enzymatic hydroxylation of 5-methylcytosine (5-mCyt) by TETs generates 5-hydroxymethylcytosine (5-hmCyt), and further oxidation results in the formation of 5-formylcytosine (5-fCyt) and 5-carboxylcytosine (5-caCyt). The AID protein is involved in the deamination of 5-mCyt to thymine and of 5-hmCyt to 5-hydroxymethyluracil (5-hmUra) [17, 18]. It was proven that 5-hmUra is also generated from thymine by TET enzymes [19]. The modified bases can be subsequently excised by thymine DNA glycosylase (TDG) via the base excision repair (BER) pathway and replaced with cytosines, resulting in the demethylation of DNA [20, 21]. It is assumed that, following excision from DNA, modified bases are released into the bloodstream and eventually appear in the urine. The presence of modifications in the urine represents the primary repair product of DNA damage in vivo and reflects the activity of repair pathways [22, 23].

Epigenetic processes may also affect the regulation of crucial genes implicated in drug response and targets [24, 25]. It has been shown that DNA methylation might regulate important signaling pathways involved in therapy resistance in different cancer types [26].

In this study, we investigated the impact of chemotherapy on the epigenome and its relationship with therapy outcomes. We analyzed whether the key molecules involved in active DNA demethylation in leukocytes and urine during systemic treatment and in the tumor tissue collected after NACT may predict the outcome of breast cancer, influencing DFS and overall survival (OS) at the 6-year follow-up after the completion of radical treatment.

To our knowledge, this is the first study to comprehensively analyze the compounds involved in active DNA demethylation in leukocytes, tumor tissues, and metastatic regional lymph nodes of breast cancer patients during NACT.

## Methods

### Study group

The study included 71 women with diagnosed breast cancer who were qualified to undergo NACT at the Chemotherapy Outpatient Clinic of the Oncology Center in Bydgoszcz from 2016 to 2018. Women included in the study were between 34 and 79 years of age. The median age was 53 years. The diagnosis was based on a histopathological examination of the material collected via core needle biopsy (CNB). Standard immunohistochemical tests were performed, including the expression levels of estrogen and progesterone receptors, the Ki-67 marker, and the expression of the HER2 receptor. Ki-67 expression was assessed in all tumor samples before NACT. Post-treatment evaluation was performed only in tumors with partial pathological response (pPR) or no pathological response.

Breast cancer was classified into biological subtypes according to surrogate immunohistochemical definitions based on the expression of estrogen receptor (ER), progesterone receptor (PR), HER2, and the proliferation marker Ki-67. Luminal A tumors were defined as ER-positive and/or PR-positive, HER2-negative, with a Ki-67 index < 20%. Luminal B tumors were defined as ER-positive and/or PR-positive with either HER2 positivity or a Ki-67 index ≥ 20%. Luminal HER2-positive tumors were defined as ER- and/or PR-positive with HER2 overexpression. Non-luminal HER2-positive tumors were defined as ER-negative, PR-negative, and HER2-positive. Triple-negative breast cancer was defined as ER-negative, PR-negative, and HER2-negative.

NACT consisted of four cycles of chemotherapy according to the AC regimen every 21 days. Doxorubicin and cyclophosphamide were administered at doses of 60 mg/m^2^ and 600 mg/m^2^, respectively. Patients subsequently received a taxane drug (paclitaxel) at a dosage of 80 mg/m^2^ every week for 12 weeks. In addition, patients with the HER2-positive subtype, together with paclitaxel, received trastuzumab subcutaneously at a dose of 600 mg every three weeks, which was continued for one year after surgery as adjuvant treatment.

The study had a prospective–retrospective design. Biological samples were collected prospectively, while survival analyses were performed retrospectively after completion of patient follow-up.

Blood and urine samples were taken from patients four times:

- Point A—After the diagnosis of breast cancer, before NACT;
- Point B—After the AC regimen (doxorubicin and cyclophosphamide);
- Point C—After taxane therapy (paclitaxel);
- Point D—Approximately one month after surgery.

In addition, tissue samples were collected from breast cancer tumors and regional metastatic lymph nodes during surgical procedures following NACT. Only lymph nodes that remained involved after treatment were evaluated. Of the 71 included patients, 39 presented with clinically involved lymph nodes at diagnosis, and 10 of these achieved a pathological complete response (pCR) after NACT; among these, breast tissue was sampled from the original tumor site.

### Assessment of ER, PR, and HER2 status in breast cancer

ER, PR, and HER2 status were evaluated by immunohistochemistry (IHC) in accordance with the current American Society of Clinical Oncology/College of American Pathologists (ASCO/CAP) guidelines and carried out at the Department of Cancer Pathology of the Oncology Center in Bydgoszcz. Formalin-fixed paraffin-embedded (FFPE) tumor tissue blocks were sectioned at a thickness of 5 μm. and mounted on glass slides. Following deparaffinization and rehydration, antigen retrieval was performed using a heat-induced epitope retrieval (HIER) method. Immunohistochemical staining was carried out using monoclonal antibodies from Ventana: anti-HER2/neu (clone 4B5), anti-progesterone receptor (clone 1E2), and anti-estrogen receptor (clone SP1). All staining procedures were performed on the Ventana Benchmark Ultra automated platform (Roche Diagnostics) according to the manufacturer’s protocols. In addition, HER2 2 + cases were further analyzed using in situ hybridization (ISH).

### Tissue expression of Ki-67 in breast carcinoma

The expression of Ki-67 was determined at the Department of Cancer Pathology of the Oncology Center in Bydgoszcz. The IHC test was performed on FFPE tissues obtained from core biopsies and after surgical resection. Sections were cut at a thickness of 5 µm, placed on Knittel Glaser adhesive microscope slides and dried at 60 °C for 2 h. The IHC staining procedure was performed on the Dako Omnis platform with a monoclonal mouse antibody (clone MIB-1) according to the protocols recommended by the Nordic Immunohistochemical Quality Control (NordiQC). The proportion of malignant cells with positive staining for the nuclear antigen Ki-67 was evaluated quantitatively and visually via light microscopy. The Ki-67-labeling index is the percentage of cells with Ki-67-positive nuclear immunostaining.

### Leukocyte and tissue preparation

Leukocytes were isolated from heparinized blood samples with Histopaque 1119 (Merck KGaA, Germany) according to the manufacturer’s instructions and stored at -80 °C until analysis.

Frozen tissues obtained from patients with breast cancer (tumor tissues and regional metastatic lymph nodes) were transferred to tubes containing ceramic beads (1.4 mm), and 1 mL of phosphate buffered saline (PBS) (Biomed Lublin S.A., Poland) was added to each tube. The samples were homogenized via a Bead Ruptor Elite Homogenizer (OMNI International, USA). After homogenization, the samples were placed on ice, transferred to new tubes and used for isolation and determination of DNA modifications.

### DNA isolation and enzymatic hydrolysis to deoxyribonucleosides

Leukocytes and tissue homogenates were diluted in ice-cold buffer B (10 mM Tris–HCl (Merck KGaA, Germany), 5 mM Na_2_EDTA (Merck KGaA, Germany) and 0.15 mM deferoxamine mesylate (Merck KGaA, Germany), pH 8.0). SDS (Merck KGaA, Germany) solution was added (to a final concentration of 0.5%), and the mixture was gently mixed via a polypropylene Pasteur pipette. The samples were incubated at 37 °C for 30 min. Proteinase K (Merck KGaA, Germany) was added to a final concentration of 4 mg/mL, and the mixture was incubated at 37 °C for 1.5 h. The mixture was subsequently transferred to Phase Lock Gel Light probes, and proceeded according to the manufacturer’s instructions. The supernatant was treated with three volumes of cold 96% (v/v) ethanol to precipitate high-molecular-weight nucleic acids. The precipitate was removed with a plastic spatula, washed with ethanol and dissolved in Milli-Q grade deionized water. The samples were mixed with 200 mM ammonium acetate containing 0.2 mM ZnCl_2_, pH 4.6 (1:1). Nuclease P1 (100 U, New England Biolabs) and tetrahydrouridine (Merck KGaA, Germany), 10 μg/sample, were added to the mixture and incubated at 37 °C for 3 h. Subsequently, 10% (v/v) NH_4_OH and 6 U of shrimp alkaline phosphatase (rSAP, New England Biolabs) were added to each sample and incubated for 1.5 h at 37 °C. Finally, all the hydrolysates were ultrafiltered prior to injection to eliminate macromolecular compounds, using AcroPrep Advance 96-Well Filter Plates 10 K MWCO (Pall Corporation, USA) and centrifugation at 800 × g for 60 min at 4 °C.

### Determination of epigenetic modifications in DNA isolated from leukocytes and tissues

The analyses were performed via a method described earlier by Gackowski et al. and Starczak et al. [27, 28]. The DNA hydrolysates were spiked with a mixture of internal standards at a volumetric ratio of 4:1 to a final concentration of 50 fmol/µL: [D_3_]-5-(hydroxymethyl)-2ʹ-deoxycytidine, [^13^C_10_, ^15^N_2_]-5-formyl-2ʹ-deoxycytidine, [^13^C_10_, ^15^N_2_]-5-carboxyl-2ʹ-deoxycytidine, [^13^C_10_, ^15^N_2_]-5-(hydroxymethyl)-2ʹ-deoxyuridine. Chromatographic separation was performed with a Waters ACQUITY 2D-UPLC system with a photodiode array detector for the first dimension of the 2D-chromatography (used for quantification of the unmodified deoxyribonucleosides and 5-methyl-2ʹ-deoxycytidine) and a Xevo TQ-XS tandem quadrupole mass spectrometer (used for the second dimension of the 2D-chromatography and to analyze 5-hmdC from the first dimension in the positive mode to assure better ionization at higher acetic acid concentrations). The at-column-dilution technique was used between the first and second dimensions to improve the retention of the trap/transfer column. The following columns were used: a Waters CORTECS® T3 column (150 mm × 3 mm, 1.6 µm) with a precolumn for the first dimension, a Waters XSelect C18 CSH (100 mm × 2.1 mm, 1.7 µm) for the second dimension and a Waters XSelect C18 CSH (20 mm × 3 mm, 3.5 µm) column as a trap/transfer column. The chromatographic system was operated in heart-cutting mode, indicating that selected fractions of the effluent from the first dimension were loaded onto the trap/transfer column by 6-port valve switching, which served as the “injector” for the second dimension of the 2D-chromatography process. The flow rate for the first dimension was 0.5 mL/min, and the injection volume was 2 µL. Separation was performed with gradient elution for 10 min using a mobile phase of 0.05% acetic acid (A) and acetonitrile (B) (0.7–5% B for 5 min, column washing with 30% acetonitrile and re-equilibration with 99% A for 3.6 min). The flow rate for the second dimension was 0.3 mL/min. The separation was performed with gradient elution for 10 min using a mobile phase of 0.01% acetic acid (A) and methanol (B) (1–50% B for 4 min, an isocratic flow of 50% B for 1.5 min, and re-equilibration with 99% A until the next injection). Collision-induced dissociation was obtained using 6.0 argon at 3 × 10^–6^ bar pressure as the collision gas. The transition patterns for all the analyzed compounds and the specific detector settings were determined using the MassLynx 4.2 IntelliStart feature set in quantitative mode to ensure the best signal-to-noise ratio and a resolution of 1 at MS1 and 0.75 at MS2. All samples were analyzed with three to five technical replicates, of which the technical mean was used for further calculation. The quantities of canonical deoxynucleosides were determined by UV detection at 260 nm for 2ʹ-deoxythymidine (dT) and at 280 nm for 2ʹ-deoxyguanosine (dG) and 5-mdC. The total deoxynucleosides amount (dN) was calculated as the doubled sum of dT and dG, which served as the reference for expressing the quantities of modified deoxynucleosides.

### Determination of the level of epigenetic modifications in urine

Urine samples were collected into polypropylene containers without preservatives, within 2 h of collection, aliquoted into polypropylene tubes, and stored at − 80 °C until analysis. Sample preparation and chromatographic analysis were performed as previously described [29] with some modifications. After thawing, urine samples were spiked with a mixture of internal standards at a 4:1 volumetric ratio and centrifuged using a filtration plate equipped with a modified polyethersulfone membrane (10 kDa MWCO) at 800 × g for 20 min. The 2D-UPLC − MS/MS system consisted of a gradient pump and autosampler for one- dimensional chromatography, and a gradient pump and tandem quadrupole mass spectrometer with a UNISPRAY ion source for two-dimensional chromatography. Both systems were coupled with a column manager equipped with two programmable column heaters and two 2-position 6-port switching valves. The at-column dilution technique was used between the first and second dimensions to improve retention in the trap/transfer column. The sample molecules were then adsorbed to the packing material as very narrow bands that could be eluted with well-resolved, small-volume peaks. A diluting stream of water (0.5 mL/min) was pumped with a Waters 515 isocratic pump and mixed with the first-dimension column effluent using a UPLC low-dead-volume tee valve. The following columns were used: CORTECS UPLC T3 column (1.6 µm, 3 mm × 150 mm) with a CORTECS T3 VanGuard precolumn (1.6 µm, 2.1 mm × 5 mm) for the first dimension, a Waters ACQUITY UPLC CSH C18 column (1.7 µm, 2.1 mm × 100 mm) for the second dimension, and a Waters XSelect CSH C18 column (3.5 µm, 3 mm × 20 mm) as the trap/transfer column. The chromatographic system was operated in heart-cutting mode, which means that selected portions of effluent from the first dimension were loaded onto the trap/transfer column by 6-port valve switching, which served as an “injector” for the second dimension of the chromatography system. Mass spectrometric detection was conducted with a Waters Xevo TQ-S tandem quadrupole mass spectrometer equipped with a UniSpray ionization source. The following common detection parameters were used: source temperature, 150 °C; nitrogen desolvation gas flow, 1000 L/h; nitrogen cone gas flow, 150 L/h; desolvation temperature, 500 °C; and nebulizer gas pressure, 7 bar. Collision-induced dissociation was obtained with argon (6.0 at 3 × 10^–6^ bar pressure) as the collision gas. The instrument response to all the compounds was optimized by the infusion of 10 µM genuine compounds dissolved in water (10 µL/minute) in the mobile phase A stream via the mass spectrometer fluidics system operating in the “mixed” mode using MassLynx 4.1 software IntelliStart feature. The chromatographic system was operated with MassLynx 4.1 software from Waters Corporation, USA. Quantitative analyses were performed using the TargetLynx application. All the samples were analyzed with three to six technical replicates. Urinary levels of modified nucleosides were normalized to creatinine concentration to account for variations in urine dilution and were expressed as nmol/mmol creatinine.

### Method for determining the creatinine concentration in urine

The urine samples were diluted tenfold with deionized water, transferred to an AcroPrep 10 kDa molecular weight cut-off filtration plate (Pall Corporation, USA) and centrifuged at 800 × g for 10 min at room temperature. The resulting filtrates were sealed with pierced-seal plate foil (VWR, USA) and subjected to chromatographic analysis. To determine the creatinine concentration in urine, a calibration curve was prepared using creatinine standards with concentrations of 0.1105 mmol/L, 0.221 mmol/L, 0.442 mmol/L, and 0.884 mmol/L. The creatinine concentration was determined via high-performance liquid chromatography with UV detection (HPLC–UV) from Waters Corporation, USA. The samples were separated on a Supelcosil C18 Phenomenex column (250 mm × 4.60 mm) with a particle size of 5 µm and a Phenomenex Security Guard Cartridges HILIC precolumn (4 × 3.00 mm). The mobile phase was 0.02 mol/L phosphate buffer (KH₂PO₄) with 2% methanol, pH = 6.5. The flow rate was 1 mL/min, and the injection volume was 2 µL. The column thermostat was set at 30 °C. The effluent was monitored with a photodiode array detector at 236 nm and analyzed with Empower software.

### Gene expression analysis

The isolated leukocytes and tissues were stored at − 80 °C until analysis. RNA isolation from leukocytes was carried out with a PAXgene™ Blood RNA Kit (Qiagen, USA) according to the manual purification of total RNA from human whole blood collected via the PAXgene Blood RNA Tubes protocol. A GeneMATRIX Universal RNA Purification Kit was used to isolate RNA from tissues. The concentration and purity of the RNA samples were verified spectrophotometrically with a NanoDrop 2000 (Thermo Scientific, USA). The quality and integrity of total RNA were assessed via visualization of the 28S/18S/5.8S rRNA band pattern in a 1.2% agarose gel. The gel was stained with SimplySafe and visualized using the GBox EF Gel Documentation System (SynGene). 0.5 µg of total RNA from each sample (in a 20 μl volume) was used for cDNA synthesis via reverse transcription with High-Capacity cDNA Reverse Transcription Kit (Applied Biosystems, USA) according to the manufacturer’s instruction. The reaction was carried out with a Mastercycler Nexus Gradient thermocycler (Eppendorf, Germany). The reverse transcriptase reaction also included a negative control to exclude contamination with genomic DNA. RT‒qPCR was performed in accordance with the Minimum Information for Publication of Quantitative Real-time PCR Experiments (MIQE) guidelines. *TET1*, *TET2*, *TET3*, *AID,* and *TDG* were analyzed via relative quantitative RT‒PCR (RT‒qPCR) with relevant primers and short hydrolysis probes substituted with locked nucleic acids from the Universal Probe Library (UPL, Roche). The probes were labeled with fluorescein (FAM) at the 5′-end and a dark quencher dye at the 3′-end. The expression of the target genes was normalized to selected reference genes, *ACTB* (β–actin, Roche gene ID: 60), *G6PD* (glucose 6 phosphate dehydrogenase, Roche gene ID: 2539), *HMBS* (GeneID: 3145) and *TBP* (GeneID: 6908), using UPL Ready Assay. Real-time PCR mixtures (20 μL) were prepared from cDNA following the standard procedures for LightCycler 480 Probes Master (Roche, Switzerland). Aside from the proper samples, each plate also included no-template and no-RT controls. Quantitative real-time PCR was carried out with a LightCycler 480 II instrument. The standardizations of the reactions for each gene were performed to estimate the efficiency of amplification via standard curves. The primers and short hydrolysis probes used for *TET1-3*, *TDG*, and *AID* mRNA expression analysis are presented in Table 1.

**Table 1:** Primers and short hydrolysis probes used for *TETs*, *TDG*, and *AID* mRNA expression analysis

Gene	Forward primer sequence	Reverse primer sequence	UPL
*TET1*	5′-TCTGTTGTTGTGCCTCTGGA-3’	5′-GCCTTTAAAACTTTGGGCTTC-3’	#57
*TET2*	5′-GCCTTTGCTCCTGTTGAGTT-3’	5′-ACAAGGCTGCCCTCTAGTTG-3’	#38
*TET3*	5′-CACTCCGGAGAAGATCAAGC-3’	5′-GGACAATCCACCCTTCAGAG-3’	#1
TDG	5′-GAATGGAAGCGGAGAACG-3’	5′-TTGCTGTTCATTCACAACTGC-3’	#41
AID	5′- GACTTTGGTTATCTTCGCAATAAGA-3’	5′- AGGTCCCAGTCCGAGATGTA-3’	#69

### Statistical analyses

Statistical analyses were performed via PS IMAGO PRO 10.0 software (Predictive Solutions) and Statistica (TIBCO Software Inc. (2017) version 14. http://statistica.io/). The distributions of the analyzed variables were assessed using the Kolmogorov–Smirnov test (with Lilliefors correction) and the Shapiro‒Wilk test. Since the studied variables did not meet the criteria for a normal distribution, further analyses were carried out via nonparametric tests. Independent variables were assessed via the Mann‒Whitney U test to compare two groups. Friedman ANOVA with the Bonferroni post hoc correction was used for dependent variables.

The Kaplan‒Meier model was used to estimate the relationships between OS and DFS and continuous quantitative variables. Complete observations included the survival times from the end of surgical treatment to the patient’s death (OS) or the occurrence of recurrence or death (DFS). Censored observations represented the survival times of patients up to the last observation, at which point the patient was either alive (OS) or alive and without recurrence (DFS).

Differences between categorized groups were assessed using F-Cox tests robust to small sample sizes or a lack of complete observations. To evaluate the impact of continuous quantitative variables on survival, patients were stratified into two subgroups based on the cutoff values determined through receiver operating characteristic (ROC) curve analysis (Table 2), utilizing the Youden index. The results were considered statistically significant at *p* values less than 0.05.

**Table 2:** Cutoff values determined through receiver operating characteristic (ROC) curve analysis

	AUC	SE	*p* value	cutoff value
OS				
5-mdC/10^3^dN in leukocytes DNA (point A)	0.621	0.069	0.0802	8.279
5-cadC/10^9^dN in leukocytes DNA (point A)	0.648	0.08	0.0632	12.19
5-mdC/10^3^dN in tumor tissues DNA	0.88	0.077	0.0000	8.013
5-cadC/10^9^dN in tumor tissues DNA	0.761	0.134	0.0517	21.007
TDG mRNA expression in tumor tissues	0.75	0.109	0.0222	2.24
5-hmdU/10^6^dN in metastatic lymph nodes	0.699	0.124	0.1081	0.42
TET1 mRNA expression in metastatic lymph nodes	0.744	0.123	0.0478	0.12
DFS				
5-mdC/10^3^dN in leukocytes DNA (point A)	0.592	0.077	0.2323	8.29
5-cadC/10^9^dN in leukocytes DNA (point A)	0.716	0.086	0.0127	11.61
5-mdC in urine [nmol/mmol creatinine]	0.745	0.121	0.0433	0.882
5-cadC/10^9^dN in tumor tissues DNA	0.7	0.127	0.1164	21.007
TET1 mRNA expression in tumor tissues	0.733	0.097	0.0160	0.29
TET3 mRNA expression in tumor tissues	0.743	0.099	0.0140	0.12
TDG mRNA expression in tumor tissues	0.778	0.091	0.0022	2.24
5-hmdC/10^3^dN in metastatic lymph nodes	0.754	0.112	0.0236	0.17
5-hmdU/10^6^dN in metastatic lymph nodes	0.779	0.095	0.0034	0.239
TET3 mRNA expression in metastatic lymph nodes	0.773	0.126	0.0643	0.19

## Results

### Patient characteristics

The mean age of the study cohort at diagnosis was 55.8 years. According to the TNM classification, the most common clinical stage was IIA (56.3%), and 54.9% of patients had regional lymph node involvement (Table 3).

**Table 3:** Clinical characteristics of breast cancer patients

Biological subtype	Total	Luminal A	Luminal HER2( +)	Luminal B	Nonluminal HER2( +)	Triple negative
Number of patients	71	2	19	25	11	14
Mean age	55.8	55.5	50.5	55.2	57.4	59.3
Clinical stage	1	IA-0	IA-0	IA-1 (4%)	IA-0	IA-0
	40	IIA-0	IIA-13 (68.4%)	IIA-9 (36%)	IIA-9 (81.8%)	IIA-9 (64.2%)
	3	IIB-0	IIB-1 (5.2%)	IIB-1 (4%)	IIB-1 (9%)	IIB-0
	9	IIIA-1 (50%)	IIIA-2 (10.5%)	IIIA-5 (20%)	IIIA-0	IIIA-1 (7.1%)
	17	IIIB-1 (50%)	IIIB-3 (15.7%)	IIIB-9 (36%)	IIIB-1 (9%)	IIIB-3 (21.4%)
	1	IIIC-0	IIIC-0	IIIC-0	IIIC-0	IIIC-1 (7.1%)
Mean BMI	28.04	30.05	28.3	27.0	28.5	25.7
ER expression						
> 10%	41	2 (100%)	17 (89.4%)	22 (88%)	0	0
1–10%	3	0	1 (5.2%)	2 (8%)	0	0
1%	27	0	1 (5.2%)	1 (4%)	11 (100%)	14 (100%)
PR expression						
> 20%	33	2 (100%)	12 (63.1%)	19 (76%)	0	0
1–20%	7	0	6 (31.5%)	1 (4%)	0	0
< 1%	31	0	1 (5.2%)	5 (20%)	11 (100%)	14 (100%)
Spread to regional lymph nodes	39	2 (100%)	11 (57.8%)	14 (56%)	7 (63%)	5 (35%)
Mean Ki-67 before NACT (all patients, N = 71)	48.2%	9.0%	52.3%	49.6%	57.2%	73.2%
Mean Ki-67 after NACT (patients without pCR, N = 51)	30.2%	7.5%	26.1%	29.6%	37.0%	38.0%
Pathological response						
pCR	20	0	6 (31.5%)	5 (20%)	5 (45.4%)	4 (28.5%)
pPR	50	2 (100%)	13 (68.4%)	20 (20%)	6 (54.5%)	9 (64.2%)
No	1	0	0	0	0	1 (7.1%)
Recurrence	13	1 (50%)	3 (15.7%)	5 (20%)	1 (10.5%)	3 (21.4%)
Mean time to recurrence (months)	30.9	54.0	16.3	40.4	8.0	36.0
Deaths	10	0	2 (10.5%)	4 (16%)	1 (9%)	3 (21.4%)

In most patients (91.6%), invasive ductal carcinoma (no special type) was diagnosed, 4.2% had invasive lobular carcinoma, and the remaining cases were diagnosed as metaplastic carcinoma (1.4%), mucinous carcinoma (1.4%), and ductal carcinoma in situ with microinvasion (1.4%). A total of 64.7% of patients were diagnosed with hormone-dependent breast cancer, 42.2% with HER2-positive breast cancer, and 19.7% with triple-negative breast cancer.

Mean Ki-67 before NACT was 48.2% across all patients, decreasing to 30.2% after NACT in patients without pCR. During follow-up, 13 patients (18.3%) experienced disease recurrence, with a mean time to recurrence of 30.9 months, and 10 patients (14.1%) died.

### Changes in DNA methylation and its derivatives during chemotherapy

**Fig. 1:**
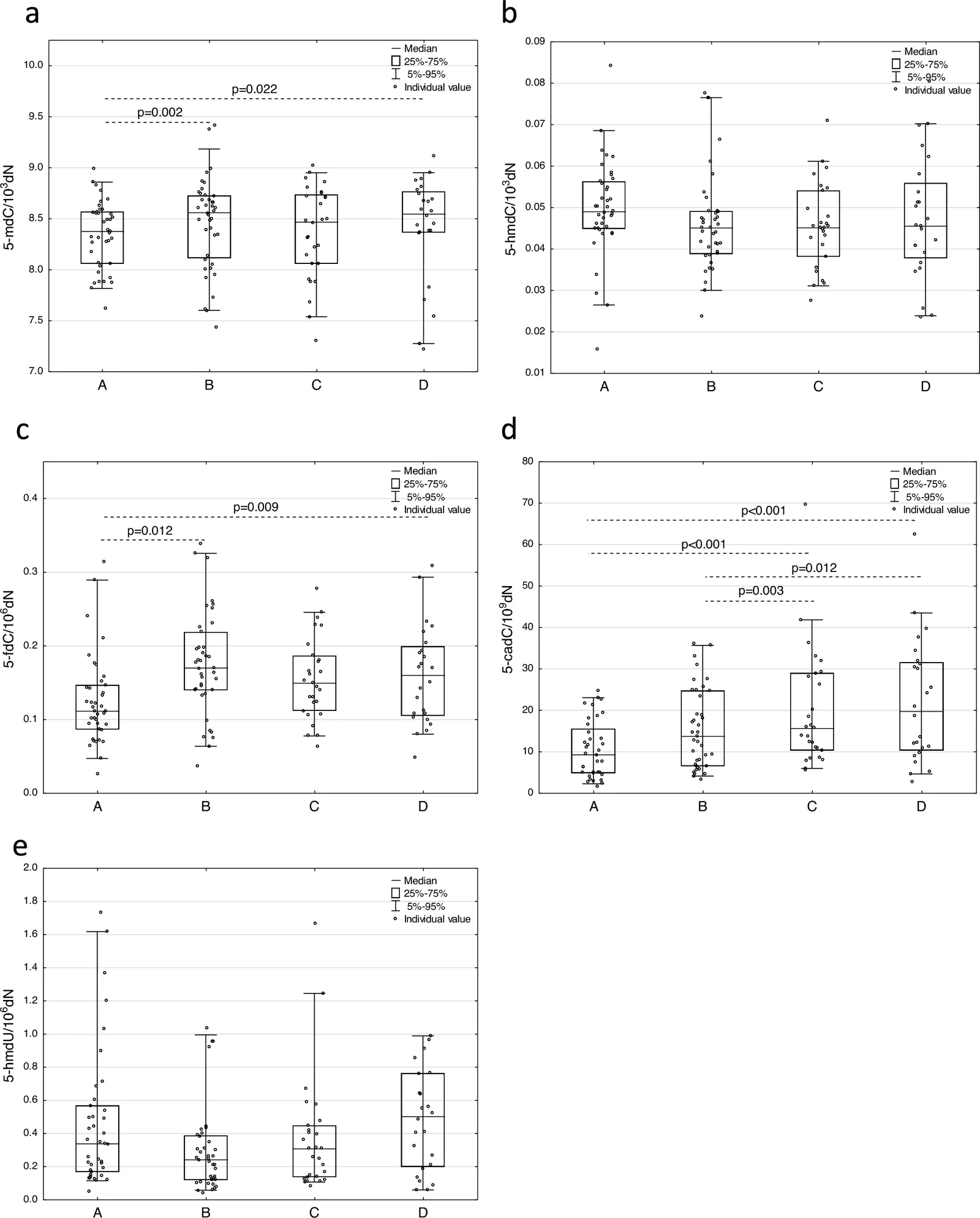
Levels of **a** 5-mdC, **b** 5-hmdC, **c** 5-fdC, **d** 5-cadC, **e** 5-hmdU in leukocytes of breast cancer patients at different time points: before NACT (A), after AC regimen (B), after taxane cycles (C), after one month after surgery (D). Data are presented as median (IQR). Only statistically significant differences (*p* < 0.05) are shown

Epigenetic modifications measured in genomic DNA were reported as 2′-deoxyribonucleosides (e.g., 5-mdC, 5-hmdC), whereas urinary biomarkers were reported both as free nucleobases (e.g., 5-mCyt, 5-hmCyt) and as nucleosides, reflecting the chemical forms detected in each biological matrix. During chemotherapy, patients were monitored for the levels of 5-mdC and its derivatives in leukocytes at four time points. The levels of 5-mdC and 5-fdC significantly increased after the administration of chemotherapy with doxorubicin and cyclophosphamide (point B), whereas no significant changes were detected following paclitaxel regimen (point C). The levels of these modifications remained elevated after the completion of chemotherapy and surgery (point D) compared with before NACT (point A). In turn, 5-cadC showed a progressive increase across subsequent blood collection points. However, no significant changes in the 5-hmdC and 5-hmdU levels were observed (Fig. 1).

**Fig. 2:**
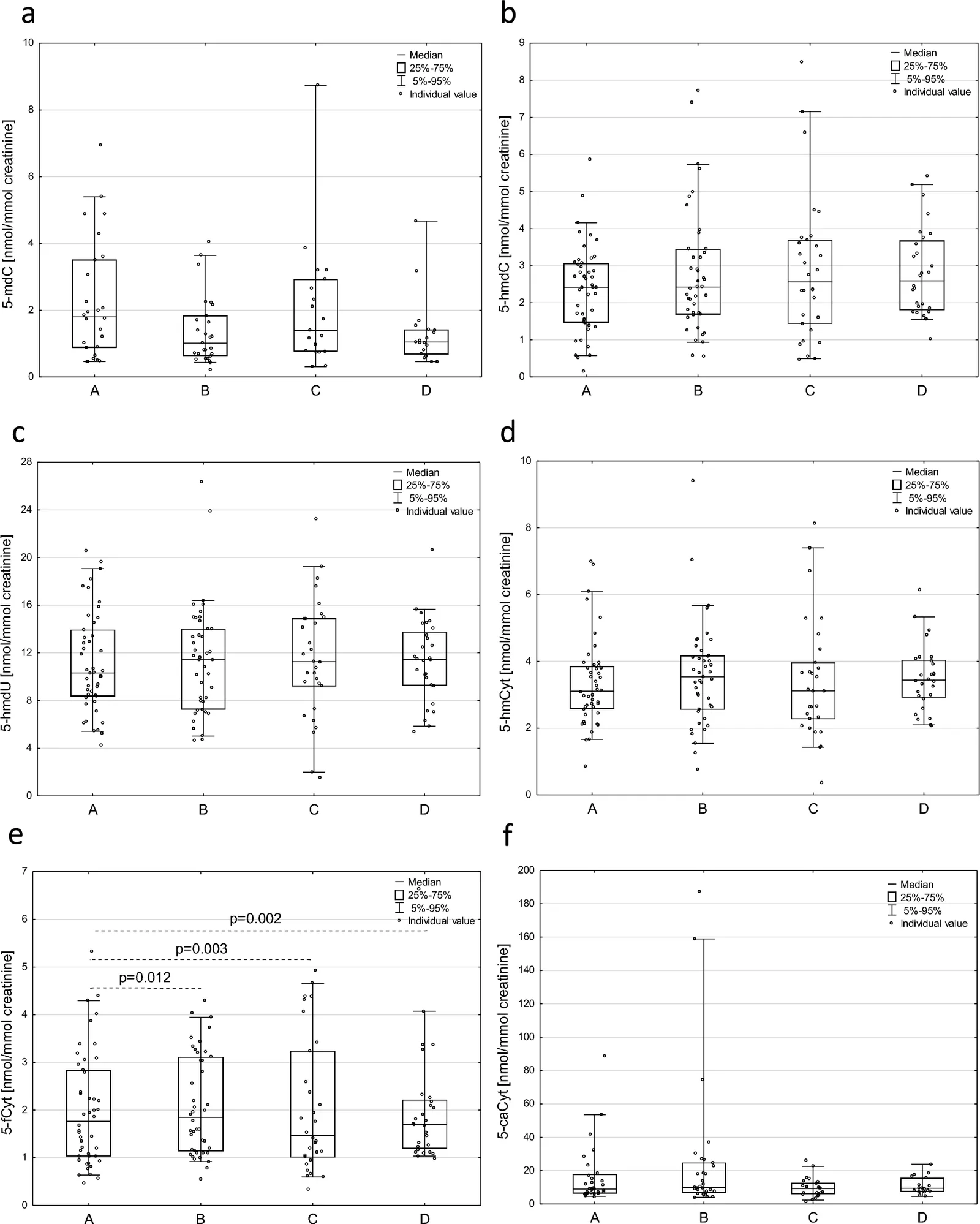
Levels of **a** 5-mdC, **b** 5-hmdC, **c** 5-hmdU, **d** 5-hmCyt, **e** 5-fCyt, **f** 5-caCyt in urine of breast cancer patients measured before NACT (A), after the AC regimen (B), after taxane cycles (C), and one month after surgery (D). Urinary levels were normalized to creatinine concentration and expressed as nmol/mmol creatinine. Data are presented as median (IQR). Only statistically significant differences (*p* < 0.05) are shown

Analysis of the levels of active 5-methylcytosine demethylation products in urine revealed statistically significant differences only for 5-fCyt. A different content of 5-fCyt was noted in urine collected after the chemotherapy AC regimen (point B), after taxane cycles (point C), and one month after surgery (point D) compared with the level before NACT (point A), corresponding to statistically significant differences between A vs B, A vs C, and A vs D (Fig. 2e).

### Expression of genes involved in active DNA demethylation

We observed a decrease in the expression of genes encoding TET proteins under the influence of chemotherapy. The most significant decrease was noted after the doxorubicin and cyclophosphamide regimens (point B). In the case of the *TET2* gene, the return of the mRNA level after treatment (point D) was demonstrated to be the level observed before chemotherapy (point A). Low *TET3* expression persisted even after the end of chemotherapy (point D).

**Fig. 3:**
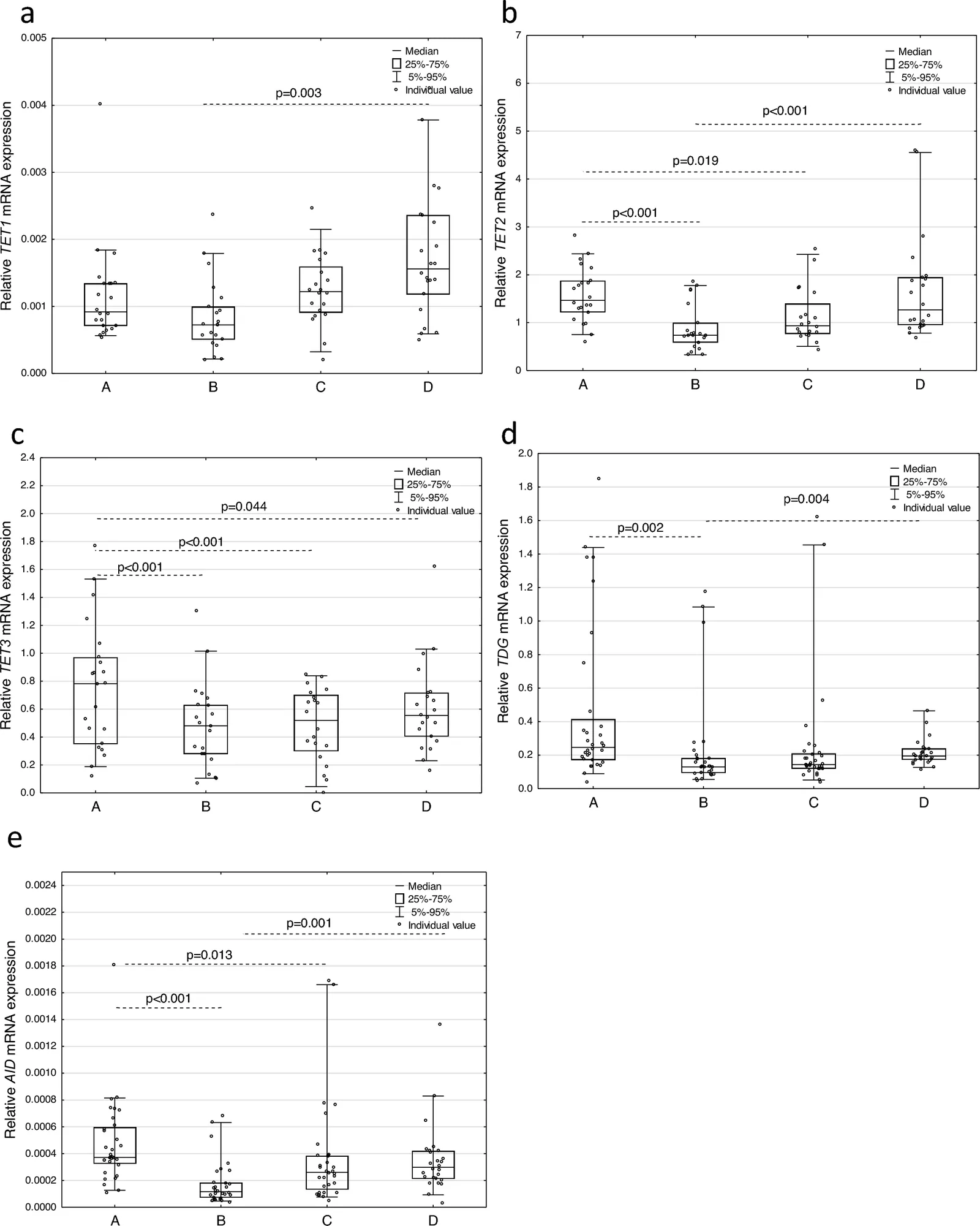
Relative mRNA expression of **a ***TET1*, **b ***TET2*, **c ***TET3*, **d ***TDG*, and **e ***AID* mRNA in leukocytes of breast cancer patients at different time points: before NACT (A), after the AC regimen (B), after taxane cycles (C), and one month after surgery (D). Gene expression levels were normalized to reference genes (*ACTB, G6PD, HMBS, TBP*). Data are presented as median (IQR). Only statistically significant differences (p < 0.05) are shown

Similarly, after chemotherapy, especially after the administration of doxorubicin with cyclophosphamide (point B), a significant decrease in the expression of genes encoding AID deaminase and TDG belonging to the BER repair enzymes was observed (Fig. 3).

### Association with clinical outcomes

**Fig. 4:**
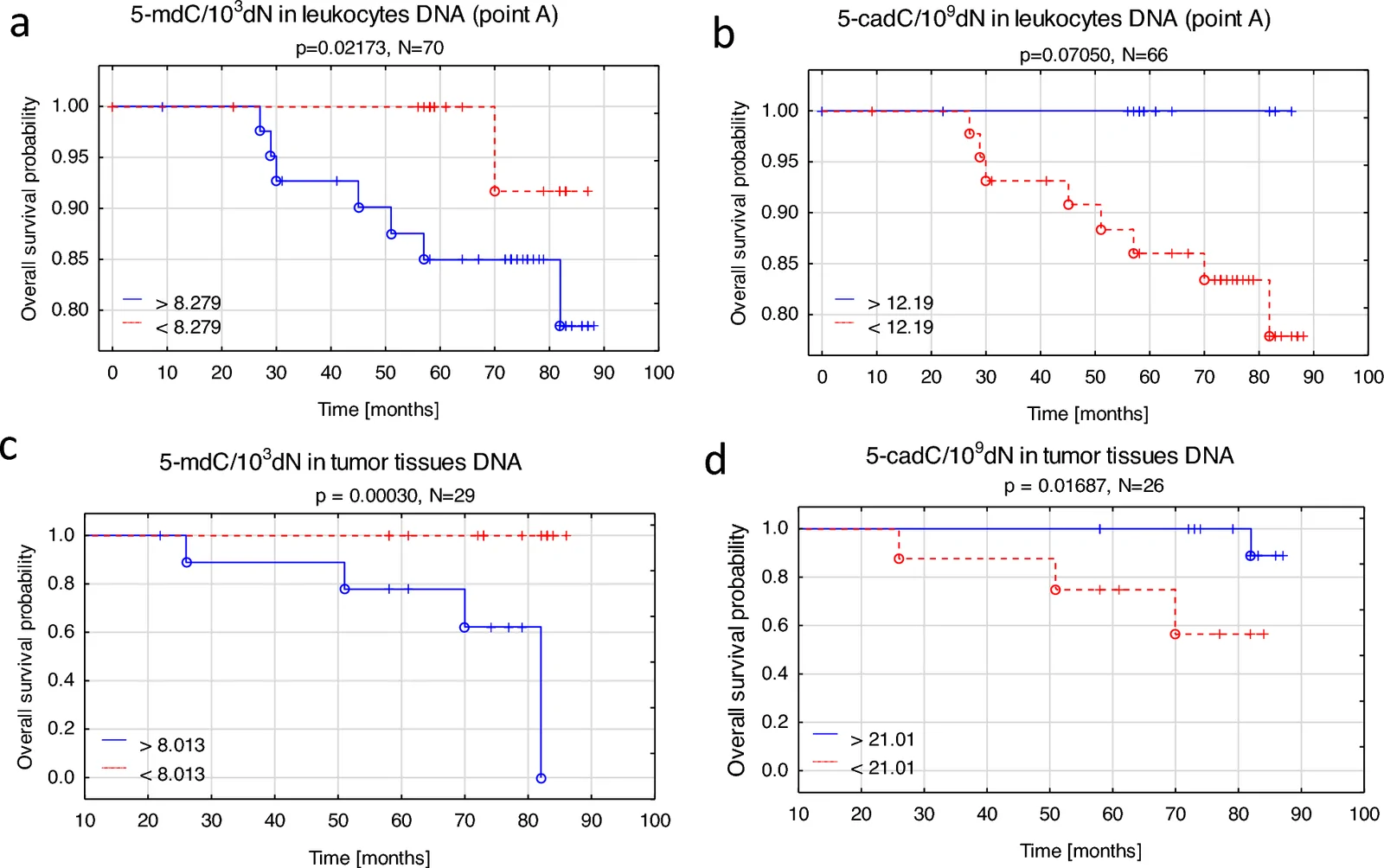
Kaplan‒Meier curves of OS in breast cancer patients stratified based on ROC-determined cutoff values of selected biomarkers. Differences between groups were assessed using the log-rank test

**Fig. 5:**
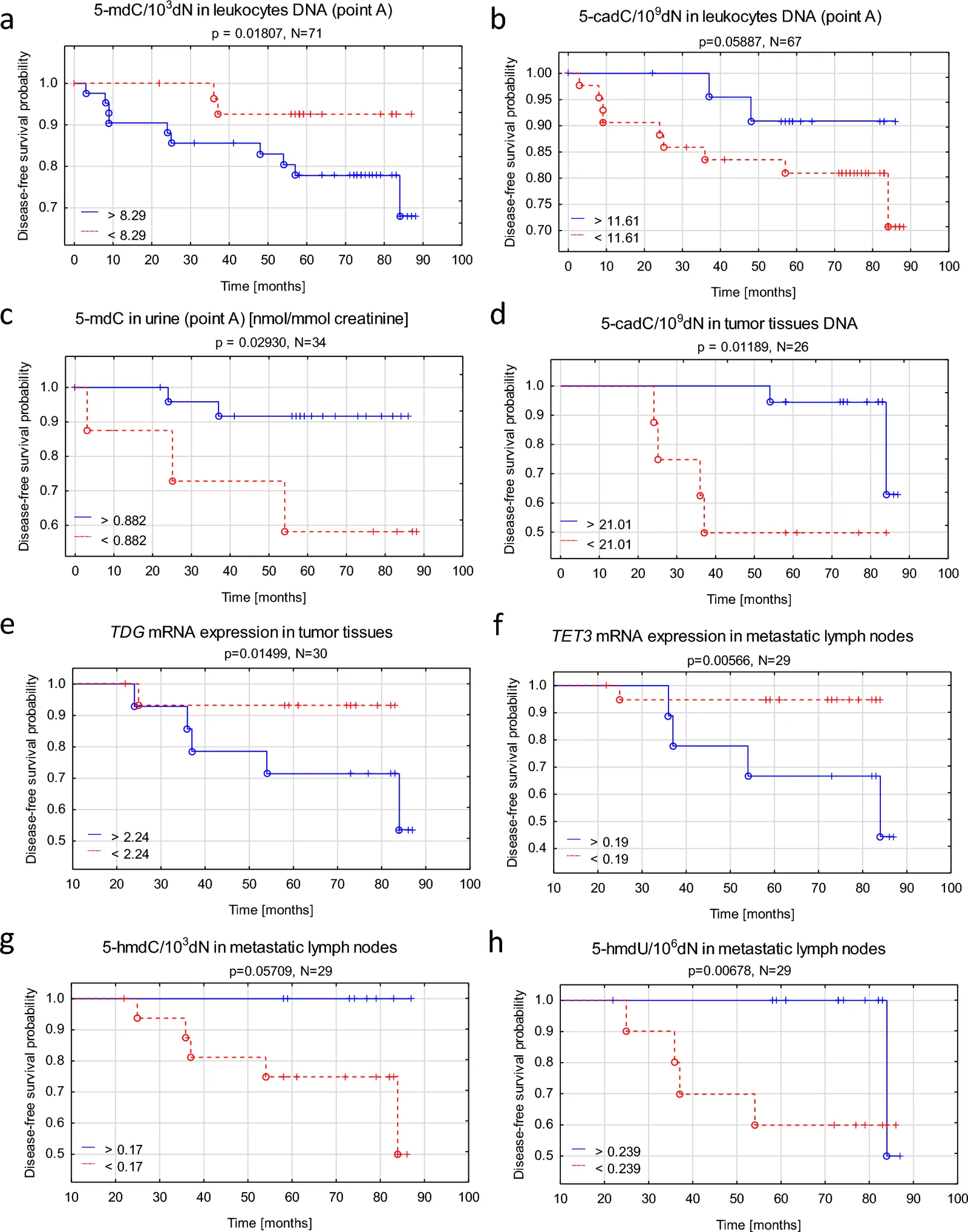
Kaplan‒Meier curves of DFS in breast cancer patients stratified based on ROC-determined cutoff values of selected biomarkers. Differences between groups were assessed using the log-rank test

To evaluate the clinical relevance of the observed epigenetic alterations, we performed Kaplan–Meier survival analysis, which revealed several associations between DNA demethylation compounds and patient outcomes (Figs. 4, 5).

OS was significantly improved in patients with lower levels of 5-mdC and higher levels of 5-cadC in leukocyte DNA collected before chemotherapy (point A). A similar pattern was observed in breast tissue obtained after NACT, in which lower 5-mdC levels and higher 5-cadC levels were associated with longer OS (Fig. 4).

DFS analysis showed that patients with lower levels of 5-mdC and higher levels of 5-cadC in leukocyte DNA at time point A had a significantly higher probability of remaining disease-free. In addition, higher urinary levels of 5-mdC measured before chemotherapy (point A) were associated with improved DFS. In tumor tissue collected after treatment, higher 5-cadC levels and lower *TDG* mRNA expression were related to a more favorable DFS. Furthermore, in metastatic axillary lymph nodes, lower *TET3* mRNA expression together with higher levels of 5-hmdC and 5-hmdU in DNA were associated with prolonged DFS (Fig. 5).

### Associations with treatment response and clinicopathological features

We also analyzed differences in the levels of compounds involved in active DNA demethylation between pPR and pCR in breast tumors after NACT and surgery (Table 4).

**Table 4:** Comparison of DNA demethylation-related parameters according to response to systemic treatment

	pPR		pCR		*p* value
	N	Median (Q1; Q3)	N	Median (Q1; Q3)	
5-fdC/10^6^dN in leukocytes DNA (point D)	19	0.1079 (0.081; 0.1849)	9	0.175 (0.1508; 0.2262)	0.0195
5-cadC/10^9^dN in breast tissues DNA	18	24.56 (18.91; 45.11)	8	107.26 (61.18; 175.39)	0.0010
5-hmdU/10^6^dN in breast tissues DNA	21	0.274 (0.2419; 0.468)	9	0.7333 (0.3486; 1.1837)	0.0490
*TET2* mRNA expression in leukocytes (point A)	51	1.362 (0.9844; 1.772)	20	1.858 (1.2525; 2.3505)	0.0298
*TDG* mRNA expression in leukocytes (point B)	30	0.1304 (0.086; 0.1817)	13	0.1819 (0.1556; 0.9886)	0.0246
*TET3* mRNA expression in leukocytes (point D)	19	0.5549 (0.3127; 0.7175)	9	0.1614 (0.0564; 0.4034)	0.0366
*AID* mRNA expression in leukocytes (point D)	20	0.0002 (0.0002; 0.0003)	9	0.0004 (0.0003; 0.0004)	0.0236

Higher levels of *TDG* expression were observed in breast cancer patients who experienced breast cancer progression during the 6 years of follow-up. In breast cancer patients who died during the observation period, a higher level of 5-mdC in cancer cells, a lower level of 5-cadC in leukocytes before NACT, and a higher level of *TET1* expression in leukocytes after NACT were observed (Table 5).

**Table 5:** Comparison of DNA demethylation-related parameters according to disease progression and survival status during follow-up

	N	Median (Q1; Q3)	N	Median (Q1; Q3)	*p* value
Progression during the follow-up period	Yes		No		
5-hmdU/10^6^dN in metastatic lymph nodes	5	0.2642 (0.1475; 0.5566)	24	0.3808 (0.2372; 0.8367)	0.0496
*TDG* expression in tumor tissues	6	2.917 (2.24; 3.966)	24	1.834 (0.7192; 2.7995)	0.0381
Death during the follow-up period	Yes		No		
5-mdC/10^3^dN in tumor tissues DNA	4	8.37 (8.07; 8.68)	25	7.86 (7.66; 8.01)	0.0162
5-cadC/10^9^dN in leukocytes DNA (point A)	8	4.82 (2.78; 8.33)	58	7.58 (4.9; 15.65)	0.0495
*TET1* mRNA expression in leukocytes (point D)	4	0.0026 (0.002; 0.0033)	24	0.0013 (0.0006; 0.0017)	0.0197

We also analyzed possible differences in the levels of epigenetic modifications, the expression of genes involved in the generation of these modifications and the clinical staging of breast cancer based on the TNM system. A significantly higher *TET3* expression in leukocytes before NACT was characteristic of more advanced stages (cT3-T4 and N1-N3) (Table 6).

**Table 6:** Comparison of DNA demethylation-related parameters according to clinical tumor size (cT) and nodal status (cN)

	N	Median (Q1; Q3)	N	Median (Q1; Q3)	*p* value
Clinical tumor size (cT)	cT1-cT2		cT3-cT4		
*TET3* mRNA expression in leukocytes (point A)	48	0.3737 (0.181; 0.823)	23	0.7092 (0.3101; 0.9702)	0.0414
*TET3* mRNA expression in leukocytes (point B)	28	0.2309 (0.0466; 0.5228)	15	0.4985 (0.2392; 0.6521)	0.0469
Clinical node involvement (cN)	cN0		cN1-cN3		
*TET3* mRNA expression in leukocytes (point A)	31	0.331 (0.1293; 0.7864)	40	0.4729 (0.3093; 0.9731)	0.0449

A higher content of 5-cadC and lower expression of the *TET3* gene in leukocytes after the completion of NACT and surgery (point C) were observed in the group with a better response to chemotherapy in metastatic axillary lymph nodes (ypN), (Table 7).

**Table 7:** Comparison of DNA demethylation-related parameters according to pathological nodal status after NACT

	ypN0		ypN1-ypN3		*p* value
	N	Median (Q1; Q3)	N	Median (Q1; Q3)	
5-cadC/10^9^dN in leukocytes DNA (point C)	21	15.85 (10.75; 28.9)	11	8.36 (5.92; 26.21)	0.0495
*TET3* mRNA expression in leukocytes (point C)	20	0.1062 (0.03; 0.4919)	12	0.5193 (0.2245; 0.7289)	0.0471

Furthermore, significant correlations were found between the levels of 5-fdC in the DNA of leukocytes, 5-cadC in DNA tumor cells, expressions of TET2, and decreased Ki-67 after NACT (Table 8).

**Table 8:** Correlations between DNA demethylation-related parameters and the Ki-67 proliferation marker

	Ki-67% before NACT	Ki-67% after NACT	decrease Ki-67 (before-after)/before
5-fdC/10^6^dN in leukocytes DNA (point B)			
r	0.182	− 0.325	0.328
p	0.2731	0.0495	0.0442
N	38	37	38
5-fdC/10^6^dN in leukocytes DNA (point D)			
r	0.418	− 0.399	0.376
p	0.0267	0.0354	0.0484
N	28	28	28
5-cadC/10^9^dN in tumor tissues DNA			
r	0.129	− 0.624	0.534
p	0.5296	0.0009	0.0049
N	26	25	26
*TET2* expression in leukocytes (point A)			
r	0.111	− 0.239	0.327
p	0.3585	0.0510	0.0057
N	71	67	70

## Discussion

Emerging evidence indicates that cytotoxic chemotherapy may influence the DNA methylation profile of breast cancer patients [30–33]. Changes in the methylation of gene promoters relevant to breast cancer have been observed in the circulating DNA of patients after NACT [34, 35]. Furthermore, genome-wide analyses of breast tumors before and after treatment with doxorubicin revealed differential methylation of hundreds of genes, including key cell cycle regulators and growth factors [10]. It has also been demonstrated that chemotherapy significantly alters the DNA methylation pattern of leukocytes in breast cancer patients [30]. A recent study indicated that methylation alterations in blood DNA are detected in patients with ovarian cancer during relapse after platinum-based chemotherapy, reflecting the changes found in tumor DNA at the time of relapse [36]. This finding suggests that DNA methylation changes may not be limited to cancer tissue but may also occur in blood leukocytes considered surrogate materials from cancer patients. In this study, we provide a comprehensive analysis of the changes in the composition of compounds involved in active DNA demethylation in nuclear blood cells. During the administration of chemotherapy drugs to patients with breast cancer, a significant increase in the level of methylated cytosine in leukocyte DNA was observed after the AC regimen (point B) and the 5-mdC level remained elevated after the completion of the chemotherapy course (point D) compared with that at point A (Fig. 1a). Similarly, in a study on a small group of breast cancer patients after preoperative chemotherapy according to schemes CMF (cyclophosphamide, methotrexate, 5-fluorouracil) and AC (doxorubicin, cyclophosphamide), a significant increase in the content of 5-mCyt in tumor tissue samples was observed compared with that in the tissues of patients without chemotherapy. Since none of these drugs (chemical substances) can directly methylate cytosine at position 5, the authors hypothesized that they act indirectly by activating DNA methyltransferases or affecting the DNA substrate [37]. High levels of 5-mCyt in DNA from patients treated with chemotherapy might result in global inhibition of protein synthesis and repression of DNA repair. Zhang et al. [38] demonstrated that doxorubicin upregulated the expression of DNMT3a, especially at concentrations that induce apoptosis in HCT116 colorectal cancer cells, and that p53 was involved in this process.

Pedersen et al. reported the loss of methylation in CpG islands and the gain of methylation outside CpG islands in genes associated with transcription factors, cell adhesion, and the immune response after NACT in 5‐year survivors. They noted that DNA methylation patterns changed before and after NACT in breast cancer cells and that these alterations induced by cytostatic drugs may be potential prognostic tools, regardless of the survival outcome of the chemotherapy regimen [33].

On the basis of the analysis of Kaplan‒Meier curves, a greater probability of DFS and OS was observed in breast cancer patients with lower levels of 5-mdC in the peripheral blood leukocyte DNA collected before chemotherapy (point A) than in patients with higher levels of this modification in leukocyte DNA (Figs. 4a, 5a). A similar result was obtained for 5-mdC levels in breast tumor tissues collected during surgical procedures following NACT. Lower levels of 5-mdC in tumor tissue were associated with a higher probability of OS (Fig. 4c). In a retrospective analysis, breast cancer patients who died during follow-up due to disease progression presented elevated levels of 5-mdC in the DNA of post-chemotherapy tumor tissue (Table 5). Our results indicate that the breast cancer cells of patients with a better prognosis after NACT exhibit lower levels of 5-mdC. This could suggest that cells with less methylated genomic DNA may be more susceptible to chemotherapy, whereas those with higher levels of methylated cytosine are more likely to survive. Therefore, higher levels of 5-mdC in DNA were observed after subsequent chemotherapy cycles. It was demonstrated that patterns of DNA methylation of genes related to cell cycle regulation differ between responders and nonresponders during NACT with doxorubicin [10]. In turn, Luo et al. [39] reported that NACT dramatically changed the methylation profiles of breast cancer tumors, and the alterations were heterogeneous between individuals, suggesting that even the same regimen could have varying effects on different patients at the epigenetic level.

Intriguingly, analyses of the associations between DFS and the levels of 5-mdC in the urine of breast cancer patients demonstrated that longer DFS was associated with higher levels of this nucleoside in the urine (Fig. 5c). The most plausible source of epigenetic DNA modifications, such as 5-fCyt, 5-caCyt, and 5-hmUra in urine, should be excised because of glycosylase(s) activity [40]. The removed bases (or deoxynucleosides) are released into the bloodstream and eventually appear in the urine. The modifications found in the urine are considered the products of DNA repair processes in the body and may reflect repair pathway activity [22].

Another source of epigenetic markers found in urine may be the processive demethylation pathway proposed by Franchini et al. [41] Specifically, 5-fCyt, 5-caCyt, and 5-hmUra can initiate processive DNA demethylation. A single initiating event, such as a specific mismatch such as 5-hmUra and uracil mispaired with guanine, can be removed via a long BER or nucleotide excision repair (NER) pathway, leading to the demethylation of numerous 5-mCyt residues at the same locus [42]. This could explain the presence of 5-mdC in urine.

Our results indicated that breast cancer patients with a better prognosis have a reduced level of 5-mdC in the DNA of peripheral blood leukocytes and an elevated level of 5-mdC in urine before NACT, which suggests that these markers could become specific prognostic indicators for this group of patients.

Similar to those of 5-mdC, significantly higher levels of 5-fdC and 5-cadC were observed after chemotherapy with doxorubicin and cyclophosphamide (point B) (Figs. 1c, 1d). Furthermore, higher levels of 5-cadC in breast cancer cells surviving chemotherapy significantly impact the extension of DFS and OS (Figs. 4d, 5d). Patients who died during the observation period presented lower levels of 5-cadC in leukocyte DNA before chemotherapy (Table 5). Additionally, a high level of 5-cadC in DNA from tumor tissues resulting in pCR to systemic treatment was demonstrated (Table 4). Therefore, a higher content of this modification in DNA indicates a better prognosis for patients. Similarly, recent research revealed that a higher level of 5-caCyt in prostate cancer cells obtained during prostatectomy was associated with an extended time to biochemical recurrence [43]. 5-MCyt is commonly considered an epigenetic marker that specifically interacts with methyl-CpG-binding proteins and contributes to the regulation of gene expression [44]. 5-FCyt and 5-caCyt, which are found at much lower levels at steady state than 5-hmCyt, were initially regarded only as transient intermediates in the process of active DNA demethylation. However, some proteins appear to bind specifically to 5-fCyt or 5-caCyt, suggesting the potential epigenetic signaling functions of these modifications as well [45]. However, recent studies have suggested that 5-caCyt may play a more direct role in the regulation of transcription. The oncogenic transcription factor Myc and its binding partner Max can specifically recognize 5-caCyt, but display a weaker binding affinity for 5-fCyt, 5-mCyt, and 5-hmCyt [46]. Additionally, 5-hmUra, which is derived from 5-hmCyt deamination by the AID/APOBEC family or thymine oxidation by TET proteins, was shown to interact with proteins involved in chromatin remodeling and transcription regulation [19]. Intriguingly, in the case of 5-hmdU, we noticed that a lower level of this modification in the DNA of metastatic axillary lymph nodes after chemotherapy is associated with shorter DFS and disease progression (Fig. 5h).

In our study, we observed that higher levels of 5-mdC, 5-fdC, and 5-cadC after the doxorubicin and cyclophosphamide cycles were accompanied by significantly lower expression levels of genes involved in the active demethylation process, namely, *TET1-3*, *TDG*, and *AID* (Fig. 3). Patients with lower *TDG* expression in cancer cells that survived chemotherapy exhibited prolonged DFS (Fig. 5e). We also detected increased *TDG* expression in tumor tissues from breast cancer patients with disease progression/recurrence during the follow-up period (Table 5). Consistently, Yang et al. reported that changes in the levels of *TET* and *TDG* mRNAs following anthracycline administration are associated with prognosis in early-stage breast cancer [47], supporting the clinical relevance of alterations in active DNA demethylation after NACT.

Importantly, the biological interpretation of the observed changes remains complex, as they may reflect both direct consequences of chemotherapy-induced DNA damage and adaptive epigenetic responses. Cytotoxic agents such as doxorubicin and cyclophosphamide induce extensive DNA damage, including oxidative lesions and DNA strand breaks, thereby activating the DNA damage response and repair pathways [48]. DNA damage has been shown to recruit DNA methyltransferases, leading to aberrant methylation patterns [36, 49], which may partly explain the observed increase in 5-mdC levels. Notably, the active DNA demethylation machinery is mechanistically linked to DNA repair processes. Oxidized derivatives of 5-methylcytosine, such as 5-fdC and 5-cadC, are intermediates of the demethylation pathway and require TDG-dependent excision via the BER system, thereby replacing them with unmethylated cytosine [50]. Disruption of BER may therefore lead to the accumulation of these intermediates rather than completion of demethylation. Recent evidence indicates that TDG and TET1 can be upregulated in chemotherapy-resistant cancer cells, promoting locus-specific DNA demethylation and pro-survival signaling [51]. The decreased expression of *TET1–3* and *TDG* observed in our study may suggest impaired demethylation capacity or disrupted coordination between DNA repair and epigenetic regulation. Alternatively, this may represent an adaptive response limiting excessive DNA processing under sustained genotoxic stress, thereby preserving genomic integrity.

Our results further revealed that the decrease in *TDG* mRNA expression may reflect inefficient excision of epigenetic modifications from DNA, contributing to the accumulation of 5-fdC and 5-cadC in the DNA of leukocytes and decreased levels of 5-fCyt in urine after chemotherapy (Fig. 1c, d and Fig. 2e). Reduced *TDG* mRNA expression may also lead to elevated levels of 5-cadC in cancer cells in tumors after chemotherapy and both these features were associated with prolonged DFS. The decreased expression of the *TDG* gene in peripheral blood leukocyte DNA observed in patients may be related to the effectiveness of NACT. From a therapeutic perspective, it is desirable that the inability of rapidly dividing cancer cells to efficiently repair DNA damage induced by antineoplastic drugs, leading to cell death, is critical for treatment effectiveness. This observation may indicate that reduced *TDG* expression limits the efficiency of BER-mediated DNA repair in cancer cells exposed to chemotherapy, thereby enhancing the cytotoxic effects of treatment. In contrast, elevated *TDG* expression in patients with disease progression may indicate enhanced DNA repair capacity, contributing to tumor cell survival and therapy resistance. For this reason, proteins involved in the BER pathway represent promising targets for new therapies using molecules that act as inhibitors of the BER [52]. It has been demonstrated that TDG expression is directly regulated by p53, indicating that the loss of p53 function may influence processes mediated by TDG, thereby adversely affecting genetic and epigenetic stability [53]. Active demethylation processes may be directly involved in drug-induced transcriptional regulation. Notably, differences in the levels of gene transcripts associated with DNA methylation after NACT may also be related to disruptions in miRNA expression. Experiments on breast cancer cell lines have shown that both doxorubicin [54] and taxanes [55] can influence changes in the expression of various miRNAs, consequently affecting the regulation of genes related to carcinogenesis. Considering that interactions with specific miRNA molecules may influence *TET* mRNA expression levels [56–58], it can be hypothesized that changes in DNA methylation after NACT may be associated with disturbances in the expression of miRNAs.

Recently, changes in the expression or activity of TET enzymes have been suggested to promote carcinogenesis, progression, and metastasis in breast cancer patients, and TET dysregulation may serve as a prognostic, diagnostic, and therapeutic biomarker in breast cancer [59]. Our analyses revealed that lower *TET3* mRNA expression in blood leukocytes collected approximately one month after the completion of systemic treatment (point D) was associated with a pCR in the breast tumor after surgery (Table 4). We also noted that higher expression of the *TET3* gene in leukocyte DNA before chemotherapy (point A) was associated with more advanced clinical breast cancer (Table 6). In addition, higher expression of the *TET3* gene in leukocytes after the completion of chemotherapy was found in breast cancer patients with a worse response to chemotherapy in ypN (Table 7). These results suggest that *TET3* mRNA expression may be considered a prognostic factor for predicting the achievement of pCR in breast tumor tissues following NACT. Recent research revealed that elevated *TET3* expression is associated with poor clinicopathological functions and poor prognosis in patients with ovarian cancer [60]. The results of other studies showed increased transcriptomic expression of *TET2* and *TET3* in peripheral blood mononuclear cells of breast cancer patients compared to healthy donors, as well as higher mRNA expression of the programmed cell death ligand-1 (*PD-L1*). It may suggest that active demethylation could be involved in upregulating the *PD-L1* gene, whose promoter was found to be hypomethylated [61]. The interaction of PD-1 on T cells and PD-L1 favors tumor cell progression by inhibiting T-cell activity. Interestingly, it has been reported that TET2 suppresses PD-L1 expression independently of its catalytic function. It recruits histone deacetylase (HDAC 1/2) to the *PD-L1* promoter, leading to the deacetylation of H3K27ac, in turn resulting in the suppression of *PD-L1* gene transcription in breast cancer cells [62]. These findings could partly explain the observed higher *TET3* expression in more advanced disease stages and poorer response to treatment in lymph nodes as well as higher *TET2* expression in leukocytes (point A) of breast cancer patients with pCR in the tumor tissues after NACT.

Ki-67 is a proliferation marker, and its level reflects the rate of cell division [63]. A decrease in Ki-67 following chemotherapy may suggest that the therapy effectively inhibits the proliferation of cancer cells. Cancer cells sensitive to chemotherapy typically show a greater decrease in Ki-67 after treatment. A significant reduction in Ki-67 may suggest a better prognosis and is associated with relapse-free survival [64]. It was also demonstrated that increased Ki-67 expression in residual disease following NACT was linked to the early onset of metastasis [65]. We identified a positive relationship between the decrease in Ki-67 following chemotherapy and the levels of 5-cadC in the DNA of tumor cells surviving chemotherapy, as well as 5-fdC in the DNA of peripheral blood leukocytes. Notably, the post-treatment decrease in Ki-67 index positively correlated with baseline (point A) *TET2* expression in leukocytes (Table 8). This association suggests that *TET2* expression levels measured before chemotherapy initiation could serve as a predictive marker of systemic treatment response, potentially forecasting decreases in tumor cell proliferation.

may indicate a potential decrease in Ki-67 after chemotherapy, even before the initiation of systemic treatment. Therefore, *TET2* gene expression in leukocytes could be considered a predictive factor for the response to systemic therapy.

Several limitations should be considered when interpreting the findings of this study. The sample size, particularly for tumor and lymph node analyses, was relatively limited, which may have reduced statistical power and necessitates cautious interpretation of these results. The absence of multivariable analyses limits the ability to account for potential confounding factors. In particular, due to the relatively small cohort size, multivariable Cox regression analysis including established prognostic factors was not performed, and therefore the independent prognostic value of the identified epigenetic markers requires confirmation in larger cohorts. The patient population was heterogeneous with respect to molecular subtype and treatment, including the use of HER2-targeted therapy (trastuzumab) in a subset of patients with HER2-positive breast cancer, which may have influenced the observed epigenetic patterns. Previous studies indicate that HER2‑positive tumors exhibit distinct DNA methylation signatures compared to other breast cancers [66] and that methylation changes are associated with response to neoadjuvant trastuzumab‑based therapy, with differential methylation observed between pCR and non‑pCR tumors [67]. Furthermore, although all patients received a backbone NACT regimen based on doxorubicin and cyclophosphamide, treatment was not fully standardized, and differences related to tumor subtype and additional therapies may have contributed to variability in epigenetic responses.

Finally, interpretation of epigenetic modifications measured in peripheral blood and urine should also consider biological sources of variability. DNA methylation profiles obtained from whole blood may be influenced by the leukocyte composition, as different immune cell populations exhibit distinct methylation patterns and their proportions may change during systemic inflammation, cancer progression, or chemotherapy-induced hematological alterations [68–70]. For urinary biomarkers, physiological factors such as renal function and urine dilution may also affect the measured concentrations of modified nucleobases and nucleosides; therefore, normalization strategies (e.g., creatinine adjustment) are commonly applied in biomarker studies [71].

Given cohort heterogeneity, non-standardized treatment, and limited follow-up, the observed associations should be interpreted as exploratory and do not imply causality.

Despite these limitations, this study has several notable strengths. The longitudinal design, with repeated sampling at multiple clinically relevant time points, allows assessment of dynamic epigenetic changes during NACT. The inclusion of minimally invasive biospecimens (peripheral blood leukocytes and urine) alongside tissue samples (breast tumor and lymph node) provides a comprehensive and translational perspective. Additionally, the simultaneous quantification of multiple intermediates of active DNA demethylation, combined with the analysis of key regulatory enzymes, represents a mechanistically informative approach.

## Conclusions

In summary, epigenetic modifications and the regulation of active DNA demethylation pathways are closely intertwined with response to NACT and clinical outcome in breast cancer. The results of our study demonstrate that among the compounds involved in active DNA demethylation, analyses of 5-mdC in leukocytes and urine—as easily accessible biological material obtained in a minimally invasive or noninvasive manner—seem to have the potential to predict prognosis after NACT for patients with breast cancer. We also observed that *TET3* expression in leukocytes was associated with treatment response and disease stage, suggesting its potential prognostic significance. Moreover, the level of *TET2* gene expression in peripheral blood leukocytes may be informative regarding the decrease in Ki-67 after chemotherapy. Nevertheless, these findings require further validation in larger and more homogeneous cohorts.

## Data Availability

The datasets used and/or analyzed during the current study are available from the corresponding authors upon reasonable request.
